# The relationship between infestation level and the welfare of cage-free laying hens experimentally infested with northern fowl mites

**DOI:** 10.1016/j.psj.2026.106984

**Published:** 2026-04-21

**Authors:** Hayley L. Sutherland, Luiz F. Brito, Amy C. Murillo, Marisa A. Erasmus

**Affiliations:** a Department of Animal Sciences, Purdue University, West Lafayette, IN 47907, USA; b Department of Entomology, University of California, Riverside, CA, USA

**Keywords:** Laying hen, Welfare, Ectoparasite, Northern fowl mite, Productivity

## Abstract

Northern fowl mites are the most economically important and damaging ectoparasite in the U.S. egg industry. The heterogeneous nature of mite infestation poses a unique welfare challenge; some hens become severely infested and others are more resistant to infestation. This study sought to characterize the relationship between mite infestation level and laying hen welfare parameters. Purebred white laying hens (replicate 1: n = 393 hens; replicate 2: n = 600 hens) were randomly allocated to cage-free floor pens (n = 40 hens/pen; 5 pens/room) in a research facility at 17 wk. Each hen was infested with 50 northern fowl mites at 24 wk. At 12, 16, 20, 24, 26, 30, and 40-41 wk, welfare assessments were conducted and each hen was assessed for the following parameters: body weight; condition of the beak, footpads, feathers (neck, back, left wing, right wing, tail, and belly); toe damage; keel bone damage (deviations and tip fractures); and mite-associated measures (mite score, skin inflammation and area of inflammation, and skin lesions). After peak infestation (30 wk), hens were sorted into infestation level groups based on their mite score using PROC HPCLUS (SAS 9.4). Body weight, inflammation area, and feather count were analyzed with PROC GLIMMIX. All other traits were analyzed with PROC LOGISTIC and odds ratios were calculated. Footpad condition, feather condition, and keel bone damage worsened with age. Toe damage improved with age. Hens that would develop high infestation levels had worse wing and tail feather condition but better belly feather condition prior to infestation. Mite population peaked 6 weeks post-infestation and declined over the remainer of the study. Higher infestation levels were associated with worse skin lesion and skin inflammation scores and tended to be influenced by the number of feathers on the vent immediately prior to mite infestation. No differences in body weight were observed despite mite infestation. Northern fowl mite infestation level exhibits differential effects on laying hen welfare, particularly feather condition, and results in reduced welfare due to lesions and inflammation.

## Introduction

As the U.S. egg industry continues to shift to cage-free egg production, ectoparasites are poised to be more problematic than ever. Northern fowl mites (**NFM**; *Ornithonyssus sylviarum* [Canestrini and Fanzago]) are already the most prevalent ectoparasite affecting North American laying hens, causing declines in productivity and reducing hen welfare (Mullens et al., 2010; Murillo and Mullens, 2017; Win et al., 2024). In commercial settings, NFM typically reside on the vent area of laying hens and will do so for their complete life cycle because of the vent’s feather structure and favorable microclimate (Mullens et al., 2010; Murillo and Mullens, 2017; Murillo et al., 2020; Gerry et al., 2026). Northern fowl mites feed on the blood of their host hens (DeVaney and Augustine, 1987), which causes hen welfare concerns centered around skin lesions, skin inflammation, and anemia (reviewed in Holquinn et al., 2024). Heavily infested hens (> 50,000 mites) are estimated to lose up to 6% of their blood volume per day from mite feedings (DeLoach and DeVaney, 1981). Skin lesions result from the mites puncturing the dermis, but the inflammation is part of the hen’s own immune response to combat infestation by thickening the skin to block capillary access (Owen et al., 2009). Though mites can migrate to various areas on the vent to avoid the inflammatory response, the overall fitness of adult mites is negatively impacted, which leads to reductions in offspring quality (Owen et al., 2009). Previous research utilizing hen welfare scoring found mite-infested hens had skin lesions, reddened skin, grey-black feathers, and decreased feather coverage (Vezzoli et al., 2016; Murillo et al., 2020; Jarrett et al., 2022). The mite population level over time is correlated with the severity of skin lesions (Murillo et al., 2020), with worse conditions found around peak infestation, prior to immune response activation (Minnifield et al., 1993). Productivity is also negatively impacted by NFM infestation, such that infested hens exhibit lower BW, reductions in egg production and quality, and inefficacies in feed conversion (DeVaney, 1979; Mullens et al., 2009; Jarrett et al., 2022).

Although the shift to cage-free egg production is aimed at affording more behavioral opportunities to laying hens (Lay et al., 2011), NFM prevalence in commercial facilities is significantly increasing as a result (Murillo and Mullens, 2016). Historical treatment methods include synthetic pesticide treatments; however, such methods have reduced efficacy due to pesticide resistance (Mullens et al., 2017) and ineffective pesticide administration (i.e. indirect application to the vent area) in cage-free housing (Murillo and Mullens, 2016). Therefore, preventing NFM infestation altogether is the preferred management method, which can be achieved by utilizing both biosecurity protocols and integrated pest management (Axtell, 1999). Continuous monitoring for pests like NFM may help reduce economic impact by enabling producers to intervene before productivity declines (Murillo and Mullens, 2016, 2020). Still, other methods of pest control and prevention are needed to enable successful mite management programs for producers (Murillo and Mullens, 2017; Holquinn et al., 2024).

A troublesome characteristic of NFM infestation in laying hens is the heterogeneity of infestation, which has a genetic basis (DeVaney et al., 1982; Owen et al., 2008; Murillo et al., 2016). The number of mites residing on a hen varies widely between individual hens despite similarities in housing and management. Parasite load often follows the Pareto principle (“80/20 rule”) that 80% of the parasites reside on 20% of the host population (Woolhouse et al., 1997). In alignment with industry needs for preventing NFM infestation, elucidating the nuances of the hen-mite relationship, particularly what makes certain hens ideal hosts, can help combat ectoparasites as cage-free production increases and traditional treatment methods lose effectiveness. Economically, the most detrimental aspect of NFM infestation is not the mites or their blood meals, but rather the metabolic cost of fighting the infestation (Mullens et al., 2009; Owen et al., 2009), which includes the hens’ immune responses. From an animal welfare standpoint, the extent to which NFM impair hen welfare is likely a function of both the infestation level and the strength of the hen’s immune response. Previous research relates infestation level to negative effects on welfare parameters like feather condition and skin lesions (Murillo et al., 2020); however, these findings were related to the overall mite population of the flock and not individual hens. Characterizing the animal welfare impacts of different infestation levels on individual hens can provide valuable information needed to further investigate why and how individual hens differ in their mite load, leading to the potential for genetically selecting hens that are more resistant to NFM. In the present study, we sought to characterize how differential northern fowl mite infestation levels affect laying hen welfare parameters. This study was part of a larger study aimed at identifying novel heritable traits associated with laying hens’ resistance and susceptibility to NFM.

## Materials and methods

This study was approved by, and all experimental procedures were conducted in accordance with, the Purdue University Institutional Animal Care and Use Committee.

### Animals and housing

This study was completed in 2 in-time replicates. At 1 d, beak-trimmed purebred white laying hen chicks (replicate 1: n = 450 birds, replicate 2: n = 630 birds) were obtained from a commercial hatchery (Hy-Line International, Dallas Center, IA, USA) and transported to Purdue University’s Animal Sciences Research and Education Center (ASREC) Poultry Unit (West Lafayette, IN, USA). All chicks were given an individual wing band ID tag during processing at the hatchery. Chicks were initially housed in cages (0.6 m x 0.6 m x 0.46 m) in either 1 (replicate 1: n = 10 chicks/cage) or 2 rooms (replicate 2: n = 5-6 chicks/cage). In replicate 1, chicks were distributed among additional cages at 6 wk (5 birds/cage) to prevent overcrowding. Cages were used for the first 8 wk of rearing due to space availability constraints. Supplemental feeders and drinkers inside the cage were available for the first week while nipple drinkers and a feeder at the front of the cage were always available. Across all life phases, birds had *ad libitum* access to water and an age-appropriate mash diet. All diets met or exceeded the NRC (1994) requirements.

After 8 wk, pullets were moved into a floor room (replicate 1: 1 room, n = 405 birds; replicate 2: 2 rooms, n = 300 birds/room) for further rearing. Each room included a raised platform with feeders and both bell and nipple drinkers. Pullets also had access to a solid floor area with wood shavings and perches. At 17 wk, hens were transferred to identical floor rooms (replicate 1: 2 rooms, replicate 2: 3 rooms) ahead of the laying phase. Hens were housed in 5 pens/room with 40 hens/pen; each pen was 2.42 × 3.56 m. Each pen featured a feeder and bell drinker suspended above a slatted floor with a waste pit below, a solid floor area with wood shavings, and nest boxes. Additional room details are reported in Jarrett et al. (2022). In addition to their wing bands, all hens were marked with non-toxic livestock marker for individual identification (Prima Tech® Retractable Marking Sticks, NEOGEN, Lexington, KY, USA). In the laying phase, the photoperiod began at 14L:10D and was gradually adjusted to 16L:8D by 30 wk.

### Amplifier hens

At 17 wk, hens (replicate 1: n = 10 hens; replicate 2: n = 20 hens) were randomly selected and transferred to Purdue University’s Centrally Managed Laboratory Animal Facilities (West Lafayette, IN, USA) in the veterinary isolation building. Hens were housed with *ad libitum* access to feed and water alongside nest boxes and perch access in a single room. Hens also had access to additional enrichment in the form of food (fruits, mealworms), hanging toys with mirrors and bells, and a swing. The photoperiod remained at 12L:12D for the entire length of the study.

Mites were sourced from infested feathers that were cut and collected from an infested hen population maintained at the University of California – Riverside Poultry Research Facility (Riverside, CA, USA) and shipped to Purdue University. Additional mites were collected from a spontaneous northern fowl mite infestation at ASREC (prior to the start of this study; therefore, not impacting the intentional infestation of the hens used in this study). At 18 wk, the amplifier hens were infested with at least 20 mites by rubbing the mite-infested feathers on each hen’s vent area and repeating this until most mites had been introduced to the host hens. At 24 wk, mites [replicate 1: ∼19,650 mites, replicate 2: ∼30,000 mites; mite numbers were estimated using the scoring system reported in Arthur and Axtell (1983)] were collected via aspirators from the amplifier hens and stored in Pasteur pipettes for immediate transport (Owen et al., 2008) to the cage-free rooms at ASREC for infestation of the flock. Amplifier hens were maintained through 31 wk to ensure the mite population was sustained.

### Mite infestation

Following mite collection from amplifier hens at 24 wk, mites were transported to ASREC to infest the experimental flock. We verified that there were no mites present on the hens prior to infestation. Approximately 50 mites were dispensed onto the base of the vent feathers anterior to the cloaca of each hen using forceps and cotton balls. All hens received the same treatment across both replicates to promote individual variation. We selected this age to infest based on DeVaney et al. (1984), which reported heavy and quickly-developing NFM populations for hens infested at 24 wk. At 25 wk, 1 wk after infestation, sequential sampling was conducted by examining 10 hens/pen (20% of the flock) for the presence of at least 1 mite on the vent area, which was confirmed for all pens in both replicates. Between the welfare assessments (described below) following mite infestation, mite scores were assessed weekly on a subsample of 13 hens/pen (33% of the flock) who were randomly selected at 27 wk (the same hens were always sampled thereafter). Mite scoring was conducted between welfare assessments to confirm that the infestation persisted throughout the trial.

### Welfare assessments

Welfare assessments were carried out at 12, 16, 20, 24, 26, 30, and 40-41 weeks of age for all hens, assessing the following outcome-based measures according to the Welfare Quality® Protocol for Laying Welfare Quality Network (2019): body weight, beak trimming, footpad condition, toe damage, feather condition (neck, back, left wing, right wing, tail, and belly), and keel bone fractures and deviations. Keel tip fractures were assessed using the scoring method described by Heerkens et al. (2016). The keel tip was assessed separately from the rest of the keel bone. An overview of the welfare scoring protocol is available in Table 1. Birds were caught and gently restrained for assessment before weighing and re-marking, then were returned to the floor. Total handling time equated to less than 2 min per bird at each assessment period. Within replicate 2 and prior to infestation at 24 wk, the number of feathers in the 4 × 6 cm area anterior to the cloaca were counted. Starting at 24 wk and continuing through 40-41 wk, birds were also assessed for the following measures associated with NFM infestation within the 4 × 6 cm area on the vent: mite score (Arthur and Axtell, 1983), skin inflammation and inflammation area (Owens et al., 2009), and skin lesions (Welfare Quality®, 2019); all scores and welfare measures are detailed in Table 1. Skin inflammation area (cm^2^) was calculated by measuring the length and width of the inflamed area using digital calipers (6 in. 3-Mode Digital Fractional Caliper, Husky®, Bristol, TN, USA). Observers were trained by the same person (H.L.S.) to score both welfare parameters and mite-associated measures. The timing of the final welfare assessment in replicate 1 was planned for 40 wk but was pushed back to 41 wk because of a summer heat wave. The final assessment of replicate 2 occurred at 40 wk as planned; therefore, the combined dataset used the final assessment age of 40-41 wk. Replicates were completed a year apart (replicate 1: September 2023 – June 2024, replicate 2: July 2024 – May 2025).

**Table 1:** Welfare assessment scoring guide (adapted from Arthur and Axtell, 1983; Owen et al., 2009; Heerkens et al., 2016; Welfare Quality®, 2019). All hens were scored at 12, 16, 20, 24, 26, 30, and 40-41 wk. Mite count, skin inflammation, and skin lesions were assessed beginning at 24 wk.

Beak condition	0	Intact beak
	1	Moderate to light treatment with moderate to no abnormalities; lower beak should not be longer than upper beak
	2	Severe abnormalities or severe trimming, with clear abnormalities or lower beak is longer than upper beak
Footpad condition	0	Feet intact, no or minimal proliferation of the epithelium, no wounds present
	1	Necrosis or proliferation of the epithelium or chronic bumblefoot with no or moderate swelling, not dorsally visible
	2	Swollen and dorsally visible
Toe damage	0	No visible wounds or missing parts
	1	Visible wounds and/or missing parts
Feather condition (neck, back, left wing, right wing, tail, and belly)	0	No or slight wear, near complete feathering where only single feathers might be lacking
	1	Moderate wear such as damaged feathers or one or more featherless areas < 5 cm in diameter at the largest extent
	2	At least one featherless area > 5 cm in diameter at the largest extent
Keel bone damage	0	No deviations, deformations or thickened sections, keel is completely straight
	1	Deviations like flattening, s-shape, or bending, or thickened section present in very slight form
	2	Deviation or deformation of keel
Keel tip fractures	0	No keel tip fracture
	1	Keel tip fracture present
Mite count	0	0 mites
	1	1-10 mites
	2	11-50 mites
	3	51-100 mites
	4	101-500 mites
	5	501-1,000 mites
	6	1,001-10,000 mites
	7	> 10,000 mites
Skin inflammation	0	Absence of inflammation: skin is normal
	1	Actively inflamed: red/raised skin, serous exudates, or intact scabs (skin inflammation area is measured)
	2	Recovering: cracked and lifted scabs, clear white/pink skin
Skin lesions	0	No lesions, only single (< 3) pecks (damage < 0.5 cm in diameter) or scratches
	1	At least one lesion > 0.5 cm but < 2 cm in diameter at largest extent or > 3 pecks or scratches
	2	At least one lesion > 2 cm in diameter at largest extent

### Statistical analyses

Statistical analyses were performed using the SAS 9.4 software (SAS Institute, Inc., Cary, NC, USA). A cluster analysis was conducted using PROC HPCLUS where hens were clustered into 3 groups based on their mite scores at the peak of infestation at 30 wk. The cutoffs were as follows: low (**LOW**, scores 0-2, 0-50 mites, n = 324 hens), medium (**MED**, score 3, 51-100 mites, n = 245 hens), and high (**HIGH**, scores 4-7, 100-10,000+ mites, n = 420 hens; Fig. 2). These infestation level groups were used as an explanatory variable as part of all statistical models.

Welfare data [condition of beak, footpads, feathers (neck, back, wings, tail, and belly), toe damage, and keel bone damage and deviations], mite count, skin inflammation, and skin lesions were analyzed using PROC LOGISTIC. The Firth bias correction for quasi-complete separation was applied when necessary (Lu, 2016). Odds ratios were calculated, which indicate the likelihood of a certain age or infestation level receiving the reference score compared to another age or infestation level. The reference score was set to 0 (ideal condition) unless otherwise specified. Odds ratios equal to 1 indicate that exposure does not affect the odds of the outcome (Szumilas, 2010). Odds greater than 1 suggest that exposure has higher odds of the outcome whereas lower than 1 has lower odds of the outcome. Results are presented with their corresponding 95% confidence intervals.

Body weight, skin inflammation area, and feather count were analyzed using PROC GLIMMIX. The results are presented as the least-square mean (lsmean) ± standard error of the estimates (SE). The main effects of skin inflammation area were log-transformed. Replicate, pen, and bird ID were included as random effects. Post-hoc analysis of pairwise comparisons was completed using Tukey’s test. Data were considered significant when P < 0.05.

## Results

Age, but not mite infestation level, influenced condition of the beak (age: Wald Χ^2^ = 23.26, P < 0.001; infestation level: Wald Χ^2^ = 2.61, P = 0.272), footpads (age: Wald Χ^2^ = 138.09, P < 0.001; infestation level: Wald Χ^2^ = 2.10, P = 0.349), toes (age: Wald X^2^ = 160.653, P < 0.001; infestation level: Wald X^2^ = 4.832, P = 0.089), keel tips (age: Wald X^2^ = 357.18, P < 0.001; infestation level: Wald X^2^ = 2.99, P = 0.224), and BW (age: F_6,6634_ = 4751.97, P < 0.001; infestation level: F_2,6634_ = 2.08, P = 0.125; interaction: F_12,6634_ = 0.91, P = 0.553). Age and mite infestation level influenced feather condition on the neck (age: Wald X^2^ = 1,382.84, P < 0.001; infestation level: Wald X^2^ = 14.60, P < 0.001), back (age: Wald X^2^ = 1,204.57, P < 0.001; infestation level: Wald X^2^ = 18.06, P < 0.001), left wing (age: Wald X^2^ = 927.68, P < 0.001; infestation level: Wald X^2^ = 77.03, P < 0.001), right wing (age: Wald X^2^ = 963.51, P < 0.001; infestation level: Wald X^2^ = 58.38, P < 0.001), tail (age: Wald X^2^ = 1,120.99, P < 0.001; infestation level: Wald X^2^ = 44.10, P < 0.001), and belly (age: Wald X^2^ = 1,870.16, P < 0.001; infestation level: Wald X^2^ = 48.50, P < 0.001), as well as keel bone deviations (age: Wald X^2^ = 206.82, P < 0.001; infestation level: Wald X^2^ = 9.03, P = 0.011), skin lesions (age: Wald X^2^ = 861.44, P < 0.001; infestation level: Wald X^2^ = 95.92, P < 0.001), and skin inflammation (age: Wald X^2^ = 59.00, P < 0.001; infestation level: Wald X^2^ = 117.86, P < 0.001).

### Beak condition

All hens were beak trimmed at hatch using an infrared laser, therefore beak condition analyses used the reference score 2 (severe abnormalities) instead of score 0 (untrimmed). Infestation level effects on beak condition within each age are presented in Table 2.

**Table 2:** Odds ratios with 95% confidence intervals (CI) for effects of infestation level within age on beak condition, footpad condition, and toe damage. Beak condition odds ratios used a reference score of 2 (severe abnormalities). Footpad condition and toe damage odds ratios used a reference score of 0 (ideal condition). HIGH = >100 mites; MED = 51-100 mites; LOW = <50 mites. Bolded values denote significance. *P < 0.05, **P < 0.01, ***P < 0.001.

**Age**	**Comparison**	**Odds ratio estimate (95% CI)**		
		**Beak condition**	**Footpad condition**	**Toe damage**
12 wk	HIGH vs. LOW	1.272 (0.918–1.762)	1.273 (0.131–12.344)	1.444 (0.871–2.395)
	HIGH vs. MED	1.200 (0.836–1.721)	0.589 (0.024–14.629)	1.309 (0.744–2.301)
	LOW vs. MED	0.943 (0.650–1.368)	0.463 (0.019–11.499)	0.906 (0.518–1.584)
16 wk	HIGH vs. LOW	1.039 (0.737–1.465)	3.809 (0.595–24.383)	1.343 (0.714–2.527)
	HIGH vs. MED	1.258 (0.869–1.822)	0.590 (0.024–14.646)	0.976 (0.459–2.078)
	LOW vs. MED	1.211 (0.823–1.782)	0.155 (0.008–2.912)	0.727 (0.343–1.540)
20 wk	HIGH vs. LOW	1.111 (0.797–1.551)	3.245 (1.008–10.444)*	1.170 (0.510–2.688)
	HIGH vs. MED	1.030 (0.715–1.485)	0.418 (0.046–3.765)	0.838 (0.310–2.261)
	LOW vs. MED	0.927 (0.634–1.355)	0.129 (0.016–1.014)	0.716 (0.261–1.963)
24 wk	HIGH vs. LOW	1.119 (0.803–1.560)	0.990 (0.485–2.021)	0.954 (0.212–4.293)
	HIGH vs. MED	1.291 (0.907–1.839)	0.649 (0.267–1.578)	2.588 (0.723–9.262)
	LOW vs. MED	1.154 (0.799–1.666)	0.656 (0.261–1.651)	2.713 (0.672–10.957)
26 wk	HIGH vs. LOW	0.998 (0.732–1.361)	1.252 (0.729–2.150)	3.220 (0.621–16.705)
	HIGH vs. MED	0.915 (0.650–1.287)	1.239 (0.690–2.226)	1.689 (0.236–12.065)
	LOW vs. MED	0.916 (0.641–1.310)	0.990 (0.548–1.789)	0.524 (0.101–2.726)
30 wk	HIGH vs. LOW	0.744 (0.545–1.017)	0.416 (0.206–0.839)*	11.555 (0.618–216.199)
	HIGH vs. MED	0.942 (0.677–1.310)	0.557 (0.275–1.126)	8.450 (0.402–177.460)
	LOW vs. MED	1.265 (0.887–1.805)	1.338 (0.570–3.138)	0.731 (0.154–3.472)
40-41 wk	HIGH vs. LOW	0.816 (0.581–1.146)	1.257 (0.795–1.986)	0.945 (0.210–4.253)
	HIGH vs. MED	1.202 (0.848–1.705)	1.039 (0.620–1.743)	0.838 (0.152–4.607)
	LOW vs. MED	1.472 (1.011–2.145)*	0.827 (0.490–1.395)	0.886 (0.147–5.345)

### Footpad condition and toe damage

The mite infestation level effects on footpad condition and toe damage within each age are presented in Table 2. Toe damage improved with age but was not different among infestation levels.

### Feather condition

The mite infestation level effects on feather condition within age are presented in Table 3. Hens were more likely to have perfect neck feather condition at younger ages. Similarly, hens were more likely to have perfect back feathers at younger ages up to 30 wk, and no age-related differences thereafter. Similarities continued for left wing feather condition; odds of having ideal left wing feather condition decreased with age. Odds of having ideal right wing feather condition decreased with age. Increasing infestation level negatively affected right wing feather condition. Tail feather condition worsened with age through 30 wk; increasing infestation level related to worse tail feather condition. Overall, belly feather condition worsened with increasing age. The HIGH hens continued to have better belly feather condition than LOW hens through 30 wk, after which belly feather condition was similar across all infestation levels.

**Table 3:** Odds ratios with 95% confidence intervals (CI) for effects of infestation level within age on feather condition of the neck, back, wings, tail, and belly areas. Feather condition odds ratios used a reference score of 0 (ideal condition). If all birds received the same score, odds ratios were not computed (N/A). HIGH = >100 mites; MED = 51-100 mites; LOW = <50 mites. Bolded values denote significance. *P < 0.05, **P < 0.01, ***P < 0.001.

**Age**	**Comparison**	**Odds ratio estimate (95% CI)**					
		**Feather condition**					
		**Neck**	**Back**	**Left wing**	**Right wing**	**Tail**	**Belly**
12 wk	HIGH vs. LOW	3.829 (0.155–94.744)	N/A	0.616 (0.453–0.839)**	0.556 (0.407–0.760)***	0.521 (0.377–0.719)***	N/A
	HIGH vs. MED	1.772 (0.035–90.279)	N/A	0.710 (0.506–0.997)*	0.699 (0.498–0.982)*	0.694 (0.491–0.982)*	N/A
	LOW vs. MED	0.463 (0.019–11.496)	N/A	1.152 (0.805–1.650)	1.257 (0.874–1.807)	1.333 (0.915–1.943)	N/A
16 wk	HIGH vs. LOW	N/A	0.418 (0.017–10.340)	0.509 (0.374–0.693)***	0.466 (0.342–0.635)***	0.471 (0.346–0.640)***	1.256 (0.025–63.858)
	HIGH vs. MED	N/A	0.590 (0.024–14.646)	0.643 (0.459–0.901)*	0.666 (0.476–0.932)*	0.667 (0.478–0.932)*	5.350 (0.216–132.557)
	LOW vs. MED	N/A	1.413 (0.028–72.046)	1.264 (0.883–1.809)	1.429 (1.001–2.040)*	1.417 (0.995–2.019)	4.259 (0.172–105.653)
20 wk	HIGH vs. LOW	1.274 (0.473–3.430)	0.668 (0.397–1.123)	0.472 (0.351–0.636)***	0.627 (0.464–0.848)**	0.772 (0.552–1.079)	1.922 (0.538–6.871)
	HIGH vs. MED	1.695 (0.628–4.577)	0.863 (0.507–1.469)	0.709 (0.514–0.979)*	0.799 (0.574–1.113)	1.214 (0.822–1.792)	1.695 (0.420–6.838)
	LOW vs. MED	1.330 (0.492–3.600)	1.292 (0.711–2.350)	1.502 (1.076–2.096)*	1.274 (0.907–1.790)	1.572 (1.056–2.340)*	0.881 (0.246–3.158)
24 wk	HIGH vs. LOW	0.822 (0.587–1.150)	0.524 (0.390–0.704)***	0.408 (0.279–0.596)***	0.430 (0.273–0.677)***	0.433 (0.225–0.831)*	2.061 (1.396–3.043)***
	HIGH vs. MED	0.965 (0.674–1.382)	0.713 (0.518–0.982)*	0.726 (0.466–1.130)	0.819 (0.472–1.418)	0.726 (0.334–1.578)	2.393 (1.589–3.604)***
	LOW vs. MED	1.175 (0.800–1.725)	1.361 (0.974–1.901)	1.779 (1.173–2.699)**	1.905 (1.143–3.174)*	1.678 (0.829–3.397)	1.161 (0.788–1.711)
26 wk	HIGH vs. LOW	0.439 (0.319–0.604)***	0.789 (0.574–1.086)	0.448 (0.222–0.903)*	0.628 (0.297–1.325)	0.622 (0.243–1.596)	1.644 (1.217–2.222)**
	HIGH vs. MED	0.505 (0.357–0.715)***	0.835 (0.590–1.183)	0.578 (0.264–1.269)	0.854 (0.359–2.027)	0.673 (0.241–1.878)	1.257 (0.909–1.738)
	LOW vs. MED	1.152 (0.818–1.621)	1.058(0.739–1.514)	1.292 (0.637–2.619)	1.359 (0.590–3.131)	1.081 (0.405–2.881)	0.764 (0.543–1.076)
30 wk	HIGH vs. LOW	0.962 (0.394–2.351)	1.428 (0.474–4.304)	0.789 (0.016–40.065)	0.788 (0.081–7.636)	0.422 (0.017–10.434)	2.228 (1.635–3.037)***
	HIGH vs. MED	1.095 (0.400–3.000)	1.349 (0.411–4.428)	0.198 (0.008–4.903)	1.794 (0.072–44.475)	0.557 (0.022–13.816)	1.647 (1.176–2.308)**
	LOW vs. MED	1.138 (0.400–3.241)	0.944 (0.251–3.554)	0.251 (0.010–6.225)	2.277 (0.092–56.473)	1.322 (0.026–67.320)	0.739 (0.525–1.041)
40-41 wk	HIGH vs. LOW	1.165 (0.550–2.467)	0.818 (0.374–1.787)	0.793 (0.016–40.311)	1.261 (0.130–12.221)	N/A	1.300 (0.974–1.736)
	HIGH vs. MED	1.236 (0.537–2.842)	1.387 (0.563–3.417)	0.198 (0.008–4.903)	0.557 (0.022–13.819)	N/A	1.044 (0.762–1.430)
	LOW vs. MED	1.061 (0.436–2.581)	1.696 (0.667–4.311)	0.250 (0.010–6.187)	0.442 (0.018–10.963)	N/A	0.803 (0.578–1.116)

### Keel bone damage

Infestation level effects on keel bone damage within age are presented in Table 4. Keel bone deviation prevalence generally increased with age. Keel tip fractures were generally more prevalent with older ages.

**Table 4:** Odds ratios with 95% confidence intervals (CI) for effects of infestation level within age on keel bone damage. Keel bone damage odds ratios used a reference score of 0 (ideal condition). If all birds received the same score, odds ratios were not computed (N/A). HIGH = >100 mites; MED = 51-100 mites; LOW = <50 mites. Bolded values denote significance. *P < 0.05, **P < 0.01, ***P < 0.001.

**Age**	**Comparison**	**Odds ratio estimate (95% CI)**	
		**Keel bone deviations**	**Keel tip fractures**
12 wk	HIGH vs. LOW	0.638 (0.426–0.955)*	1.135 (0.433–2.978)
	HIGH vs. MED	0.848 (0.556–1.294)	0.785 (0.239–2.580)
	LOW vs. MED	1.329 (0.831–2.126)	0.692 (0.206–2.327)
16 wk	HIGH vs. LOW	0.957 (0.670–1.366)	N/A
	HIGH vs. MED	.073 (0.728–1.581)	N/A
	LOW vs. MED	1.121 (0.746–1.686)	N/A
20 wk	HIGH vs. LOW	0.717 (0.508–1.012)	0.140 (0.007–2.616)
	HIGH vs. MED	0.721 (0.494–1.050)	0.557 (0.087–3.574)
	LOW vs. MED	1.005 (0.669–1.511)	3.989 (0.161–98.875)
24 wk	HIGH vs. LOW	0.863 (0.631–1.179)	0.896 (0.599–1.341)
	HIGH vs. MED	1.062 (0.761–1.483)	1.017 (0.662–1.560)
	LOW vs. MED	1.231 (0.864–1.755)	1.134 (0.718–1.792)
26 wk	HIGH vs. LOW	0.889 (0.641–1.232)	0.860 (0.570–1.298)
	HIGH vs. MED	0.745 (0.517–1.073)	1.303 (0.863–1.968)
	LOW vs. MED	0.838 (0.569–1.232)	1.515 (0.970–2.367)
30 wk	HIGH vs. LOW	0.774 (0.570–1.052)	0.917 (0.634–1.325)
	HIGH vs. MED	0.795 (0.570–1.110)	1.171 (0.798–1.718)
	LOW vs. MED	1.027 (0.721–1.464)	1.277 (0.849–1.921)
40-41 wk	HIGH vs. LOW	0.927 (0.691–1.244)	1.104 (0.815–1.497)
	HIGH vs. MED	1.133 (0.824–1.557)	1.140 (0.820–1.586)
	LOW vs. MED	1.221 (0.875–1.706)	1.032 (0.732–1.456)

### Mite-related parameters

**Fig. 1:**
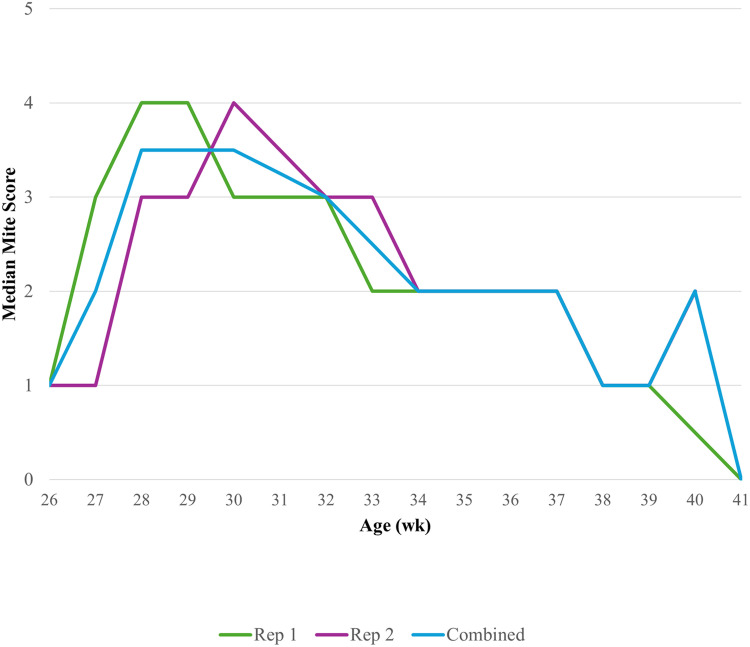
Median mite scores across the infestation period. All hens were infested with 50 northern fowl mites (NFM) at 24 wk. Mite scores collected weekly on a subsample (replicate 1: n = 130 hens, replicate 2: n = 195 hens, combined: n = 325 hens) of hens in between 26, 30, and 40-41 wk, when all hens were assessed (replicate 1: n = 393 hens, replicate 2: n = 600 hens). HIGH = >101 mites; MED = 51-100 mites; LOW = <50 mites.

**Fig. 2:**
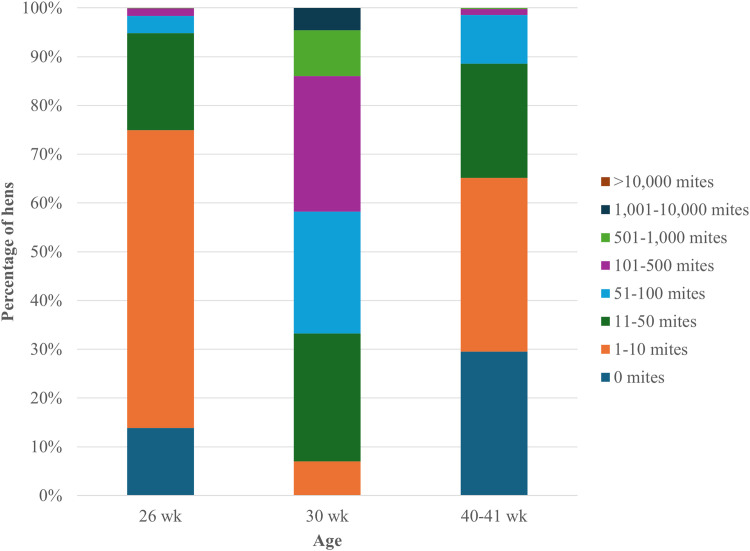
Percentage of hens with each mite score across three welfare assessment periods. Mite score was assessed in a 4 × 6 cm area on the vent, anterior to the cloaca. Hens were infested at 24 wk; the ages on the graph represent 2 wk, 6 wk, and 16-17 wk post-infestation, respectively.

Mite-related parameters were assessed beginning at 24 wk. Median mite score as tracked in the subsample of hens from 26 wk to 40-41 wk are presented in Fig. 1. The minimum mite score tracked within the subsample of hens ranged from 0 (0 mites) to 1 (1 – 10 mites). The maximum mite score tracked within the subsample ranged from 4 (100 – 500 mites) to 6 (1,001 – 10,000 mites). The proportion of total hens with each mite score from the 26, 30, and 40-41 wk welfare assessments are presented in Fig. 2. No hen received a mite score of 7 (> 10,000 mites), regardless of age or replicate. There was a stepwise increase in odds of having skin lesions alongside increasing infestation level at 30 wk (Table 5).

**Table 5:** Odds ratios with 95% confidence intervals for effect of infestation level within age on the mite-associated measures of skin lesions and skin inflammation. Mite-associated measures were assessed at 24, 26, 30, and 40-41 wk. Skin lesion and skin inflammation odds ratios used a reference score of 0 (ideal condition). If all birds received the same score, odds ratios were not computed (N/A). HIGH = >101 mites; MED = 51-100 mites; LOW = <50 mites. Bolded values denote significance. *P < 0.05, **P < 0.01, ***P < 0.001.

**Age**	**Comparison**	**Odds ratio estimates (95% CI)**	
		**Skin lesions**	**Skin inflammation**
24 wk	HIGH vs. LOW	0.606 (0.062–5.878)	N/A
	HIGH vs. MED	1.356 (0.224–8.194)	N/A
	LOW vs. MED	2.240 (0.201–24.943)	N/A
26 wk	HIGH vs. LOW	1.551 (0.996–2.413)	N/A
	HIGH vs. MED	0.958 (0.564–1.626)	N/A
	LOW vs. MED	0.618 (0.367–1.040)	N/A
30 wk	HIGH vs. LOW	0.179 (0.132–0.243)***	0.210 (0.156–0.281)***
	HIGH vs. MED	0.440 (0.332–0.600)***	0.381 (0.282–0.516)***
	LOW vs. MED	2.461 (1.768–3.427)***	1.817 (1.317–2.507)***
40-41 wk	HIGH vs. LOW	0.432 (0.320–0.584)***	0.481 (0.364–0.634)***
	HIGH vs. MED	0.593 (0.432–0.815)**	0.813 (0.602–1.099)
	LOW vs. MED	1.371 (0.967–1.944)	1.692 (1.236–2.317)**

There were no differences in skin inflammation prior to peak infestation. At 30 wk, HIGH, MED, and LOW hens all differed. Infestation level effects on skin lesions and inflammation within each age are presented in Table 5.

**Fig. 3:**
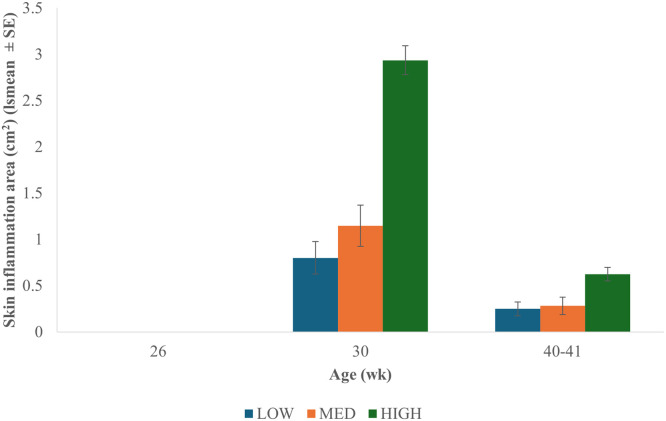
Average skin inflammation area (cm^2^). Skin inflammation area was measured in a 4 × 6 cm area on the vent area of laying hens with active skin inflammation at 26, 30, and 40-41 wk. Hens were infested at 24 wk. No skin inflammation was observed at 26 wk. There were no statistical differences because of age, infestation level, or their interaction. HIGH = >101 mites; MED = 51-100 mites; LOW = <50 mites.

Skin inflammation area did not differ because of age (F_1,542_ = 0.00, P = 0.969), infestation level (F_2,542_ = 0.69, P = 0.500), or their interaction (F_1,542_ = 0.00, P = 0.997; Fig. 3).

### Feather count (Replicate 2)

The number of feathers present on the vent prior to infestation tended to differ among infestation levels (F_2,587_ = 2.76, P = 0.064). The future HIGH hens (43.14 ± 0.56 feathers) tended to have more feathers than MED hens (40.94 ± 0.79 feathers, P = 0.061). The future LOW hens (41.86 ± 0.75) did not differ in feather count from either HIGH or MED hens.

### Body weight

**Fig. 4:**
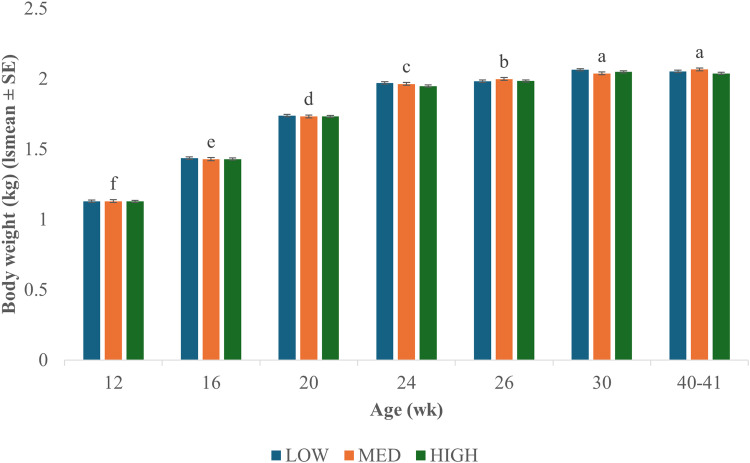
Average body weight (kg). Body weights were collected on each hen during each welfare assessment at 12, 16, 20, 24, 26, 30, and 40-41 wk. Hens were infested after assessment at 24 wk. Body weight increased with age (P < 0.001) but was not affected by infestation level (P = 0.125) or their interaction (P = 0.533). HIGH = >101 mites; MED = 51-100 mites; LOW = <50 mites. Letters indicate significant differences because of age (P < 0.05).

Body weight increased at each welfare assessment from 12 through 30 wk (Fig. 4). Body weight remained the same between 30 and 40-41 wk (P = 0.999).

## Discussion

The purpose of our study was to assess individual hen variation in NFM infestation level and the effect of relative infestation level on laying hen welfare parameters. To the authors’ knowledge, this is one of the largest NFM studies completed in laying hens to date, with individual-level data collected on close to 1,000 animals across 7 different time points; there do not appear to be other studies examining relationships among welfare measures and the severity of mite infestation level. The genetic line of hens used in this study was a purebred (pedigreed, from nucleus flocks) line, which was key to being able to examine hens’ variability in NFM susceptibility so that future research can identify heritable traits to breed hens that are more resistant to NFM.

Beak condition affects hens’ ability to preen, which correlates with their infestation level (Mullens et al., 2010). Hens with intact beaks have a sharp tip on the upper mandible, which can be used to physically remove mites through preening, resulting in more effective parasite removal (Chen et al., 2011) and lower mite loads relative to hens with intact beaks (Mullens et al., 2010; Chen et al., 2011). All hens involved in this study were infrared beak-trimmed, which uses a laser to burn the tip of the beak, which then causes the tip to slough off within 10 d (Dennis et al., 2009). In this study, beak condition scores fluctuated over time, suggesting that beak regrowth may have been occurring, which could have enabled hens to more effectively preen, thereby removing mites. Similarly, Murillo and Mullens (2016) reported substantial beak regrowth from infrared beak trimming, resulting in significantly lower mite infestation levels compared to hot-blade trimmed hens.

Footpad condition worsened with age while toe damage improved with age. At 20 wk, future HIGH hens had better footpad condition than future LOW hens. At 30 wk, infested HIGH hens then had worse footpad condition compared to infested LOW hens. Toe damage never differed among infestation levels. The toe damage primarily occurred in the pullet phase in this study and improved over time. Toe damage can be a result of barn equipment (Rørvang et al., 2019), where pullets may have injured themselves exploring their new environment. Although toe damage can also result from toe pecking, a cannibalistic behavior in laying hens (Rodenburg et al., 2009), the decrease in toe injuries over time is inconsistent with the behavioral traits of toe pecking; typically, toe pecking is positively correlated with toe damage prevalence (Gebhardt-Henrich et al., 2023). Therefore, the toe damage recorded in this study was likely a result of environmental damage and not toe pecking behavior.

Feather condition worsened with age for all measured areas. Feather damage, particularly for the wings, can be accumulated from wear-and-tear, disease, and feather pecking (Garant et al., 2022). Even though feather condition is positively correlated with feather pecking behavior prevalence, the location of feather damage gives better insight into how damage accrued. For example, back and vent feather damage is typically attributed to feather pecking (McAdie and Keeling, 2000), whereas wing feather damage is related to environmental damage. Environmental damage arises from abrasion on metal fixtures like wire floors or feeders (Hughes, 1980), but it is less severe than feather pecking damage (Leeson and Walsh, 2004a). Feather integrity is preserved through dustbathing by removing excess feather lipids (Olsson and Keeling, 2005). Impaired grooming behavior expression could negatively impact feather condition, leaving feathers more susceptible to damage.

Still, wing and tail feather condition were notably worse for future HIGH hens as seen at 12 wk and continuing through 24-26 wk. Yet, HIGH hens had better belly feather condition immediately prior to infestation at 24 wk. The physical traits of belly feathers are different than wing and tail feathers and may not accrue environmental damage in the same way. Feathers play a role in parasite defense by forming a physical barrier, blocking skin access for blood-feeding parasites (Terrill and Shultz, 2023). Although mites can affect feather condition during infestation (Heerkens et al., 2015; Vezzoli et al., 2016; Murillo et al., 2020; Jarrett et al., 2022), worsened feather condition prior to infestation likely involves underlying biological mechanisms. Keratinization disruption can alter growing feather appearance, creating “unusual” feather shapes (Leeson and Walsh, 2004a). Keratinization disruption can be a function of nutritional deficiencies; altered feather appearance has been related to deficiencies in amino acids like cystine and methionine (Wheeler and Latshaw, 1981), and minerals like zinc (Sunde, 1972). Diets between replicates were isocaloric and formulated to meet or exceed the NRC (1994) requirements, and all hens had access to the same feed; therefore, nutritional deficiencies are unlikely to have caused the differences among hens with different infestation levels. Thyroid hormones, gonadotropins, as well as estrogen and testosterone also play major roles in feather development and growth (Leeson and Walsh, 2004b). Hens with impaired metabolic processes could be more susceptible to ectoparasite infestation. Looking to wild bird literature, other research has found ectoparasite infestation levels and feather condition to be linked to changes in preening and allopreening (Goodman et al., 2020), uropygial gland size (Møller et al., 2010), climate and temperature (Møller et al., 2013), beak conformation (Bodawatta et al., 2022), and female ornamentation (Vergara et al., 2011).

Adult bird feather estimates usually fall between 7,000 and 9,000 total feathers (Leeson and Walsh, 2004a). Feathers were counted only within a 24 cm^2^ area anterior to the vent with future HIGH hens tending to have more feathers in this location compared to MED hens, and LOW hens being intermediate but not different from HIGH or MED hens. When thousands of mites can reside on a single downy feather, even small differences in the number of feathers present drastically affect the mite population size. Both feather quality and the number of feathers present can affect the level of infestation experienced by the hen.

The scoring method outlined in the Welfare Quality® (2019) protocol distinguishes feather condition scores by the presence of broken and missing feathers. Northern fowl mites need the microclimate of the vent feathers to survive and thus would not necessarily cause breakage to the feathers (De La Riva et al., 2015). Therefore, hens with better belly feather condition did harbor higher infestation levels. By the 40-41 wk assessment period, several hens received belly feather scores of 2 (bald patch greater than 2 cm in diameter), which coincided with mite scores of 0 (0 mites) or 1 (1-10 mites); simply, mites cannot survive on the vent area if there are no feathers to reside on. Although feathers may have been technically intact, the mite exuvae (molted exoskeletons), fecal material, eggs, and other miscellaneous debris would damage the quality of the feathers, which is not reflected in the scoring system used in this trial. A better assessment of feather quality, one that can account for morphological changes and not just missing feathers, is sorely needed. As mentioned before, HIGH hens at times had comparatively worse wing and tail feather conditions but better belly feather conditions. Because the feathers have different characteristics, utilizing the same scoring system for both may not accurately capture differences. Future research involving NFM and feather condition should consider a scoring system that accounts for cleanliness and other factors than simply broken or missing feathers. Vezzoli et al. (2016) used a binary scoring system of white versus grey/black feathers, which may be more applicable when assessing feather condition for NFM-infested hens. Similarly, Murillo et al. (2020) assessed soiled vent feathers independently of feather loss, such that feather condition was consistently normal but soiled feathers increased with NFM infestation level.

Keel bone damage worsened with age. Within age, future HIGH birds had more keel bone deviations at 12 wk of age compared to future LOW birds, but this difference disappeared after 12 wk. There were no other differences in keel bone deviations or tip fractures among the infestation level groups at any age. Jarrett et al. (2022) reported worsened keel condition over time in infested Tetra Brown laying hens compared to control hens, but this was an association rather than a direct cause of infestation. Garant et al. (2022) found no differences in keel bone damage for hens with artificial feather damage purposefully caused by researchers clipping wing feathers.

Mite infestation can interplay with environmental complexity to introduce additional challenges to laying hens. Laying hens have been noted as clumsy navigators (Moinard et al., 2004), which leads to an increase in bone damage. Infestation may exacerbate this issue by causing chronic discomfort from mite feeding, which may affect hens’ ability to safely navigate their environment without crashing. Previous research on the nocturnal behaviors of NFM-infested laying hens showed increased activity, less dozing, and overall fragmented behaviors overnight, which likely has negative impacts on hen welfare (Jacobs et al., 2019). This supports the idea that mites cause irritation and restlessness (Jacobs et al., 2019; Jarrett et al., 2022). Blood-feeding ectoparasites can induce histamine responses, causing itchy sensations (Berriatua et al., 2001). Our study used cage-free floor pens with no vertical complexity beyond a 15 cm ledge separating the slatted floor from the litter area. Additionally, perches were only present during the pullet phase and not the laying phase due to space limitations.

Infestation level was estimated by combining our 26, 30, and 40-41 wk sampling points on all hens with the interim mite scoring on a subsample of hens at 25, 27-29, and 31-39 wk. Northern fowl mite infestations peak between 3 and 6 weeks after infestation is first introduced (Murillo and Mullens, 2017), which coincides with our study’s population tracking. The decline in infestation is primarily due to the hens’ immune response (Owen et al., 2009; Murillo and Mullens, 2017), which takes 4-6 weeks to fully activate after infestation begins, therefore infestation levels were lower at 40-41 wk. In replicate 1, a heat wave during 40 wk delayed our final sampling time point, which could have contributed to lower mite scores. The microclimate on the hens’ vents were likely warmer during this period, which increases mite mortality and further reduces the population.

Mites were counted in a 4 × 6 cm area on the vent, anterior to the cloaca, following the methods reported by Owen et al. (2009). This area is where the most mites are concentrated (Matthysse et al., 1974) because of the feather structure providing a nest-like microclimate (Holquinn et al., 2024) and increased difficulty for hens to preen this area when beak trimmed (Chen et al., 2011). The mite population needed to be estimated between welfare assessment time points to ensure infestation prevailed over time. However, time constraints limited both the number of hens and the body locations that could be assessed. The mite scoring system, first introduced by Arthur and Axtell (1983), uses a logarithmic scale to visually estimate the mite population, which was compared to manual counting. The visual estimation was up to 92% lower than the manual count completed under a microscope, though the manual counting was extrapolated (Arthur and Axtell, 1983).

Mullens et al. (2009) identified infestation levels above 100 mites (mite score 3) as the economic threshold of NFM infestation, where economic losses can range from 7 to 10 cents per hen over a 10 wk period of high mite infestations. Forty-two percent of hens in this study reached infestation levels above 100 mites on the vent area, yet BW impacts because of infestation level were not observed. Additionally, the experimental hens are purebred and not a commercial (crossbred) strain, so productivity is expected to be lower relative to crossbred strains, which benefit from heterosis effects. DeVaney (1979) found 5-15% reductions in egg production in Ideal 236 White Leghorn hens because of NFM infestation. Jarrett et al. (2022) found that both infested and uninfested Tetra Brown hens outperformed the productivity reported by their management guide. The burden of NFM infestation lies in the hen’s immune response to the infestation (Mullens et al., 2009), which results in varied effects on productivity. Regardless, infested hens experience worse welfare.

One unexpected event in this study resulted in some early-life mortality in replicate 1 and cataract development in both replicates 1 and 2. The birds’ symptoms were consistent with avian encephalomyelitis (AEV) upon veterinary examination. The nature of AEV is that of a viral infection affecting the central nervous system. There is no research suggesting cross-reactivity between NFM infestation and AEV.

In this study, laying hens were infested with the same number of NFM to start and differentiated over the infestation period. Approximately 58% of hens maintained mite levels below 100 mites on the vent area throughout the infestation period. Welfare parameters differed among infestation levels, particularly for feather condition, skin lesions, and skin inflammation. Hens that would harbor higher infestation levels tended to have more feathers on the vent, had better vent feather condition, and would go on to have more skin lesions and more active skin inflammation compared to hens with lower infestation levels. By characterizing the individual laying hen welfare impacts of differential NFM infestation levels, we can begin to unravel traits associated with ectoparasite resistance and resilience, opening the door to genetic selection of resistant laying hens. Developing resistant genetic lines prevents the negative effects of NFM on productivity and welfare without contributing to pesticide resistance, thereby helping to safeguard egg production. To further examine the nuances of the laying hen-NFM relationship, future research will characterize behavioral differences as they related to infestation level and genetic variability of NFM resistance. As the U.S. egg industry continues to shift to cage-free egg production, more information on ectoparasite prevention is needed to preserve laying hen welfare.

## CRediT authorship contribution statement

**Hayley L. Sutherland:** Writing – original draft, Project administration, Formal analysis, Data curation. **Luiz F. Brito:** Writing – review & editing, Resources, Methodology, Funding acquisition, Conceptualization. **Amy C. Murillo:** Writing – review & editing, Resources, Methodology, Funding acquisition, Conceptualization. **Marisa A. Erasmus:** Writing – review & editing, Supervision, Resources, Project administration, Methodology, Funding acquisition, Data curation, Conceptualization.

## Disclosures

The authors declare that they have no known competing financial interests or personal relationships that could have appeared to influence the work reported in this paper.
